# Reply to the Letter to the Editor: Radiation exposure and screening yield by digital breast tomosynthesis compared to mammography—results of the TOSYMA trial breast density related

**DOI:** 10.1007/s00330-025-11803-x

**Published:** 2025-08-19

**Authors:** Alexander Sommer, Stefanie Weigel, Hans-Werner Hense, Elke Nekolla, Veronika Weyer-Elberich, Walter Heindel

**Affiliations:** 1 Clinic for Radiology and Reference Center for Mammography Münster, University of Münster and University Hospital Münster, Münster, Germany; 2 Institute of Epidemiology and Social Medicine, University of Münster, Münster, Germany; 3 Department of Medical Radiation Protection, Federal Office for Radiation Protection, Neuherberg, Germany; 4 Institute of Biostatistics and Clinical Research, University of Münster, Münster, Germany

We thank Rongbin Jiang and Ruohui Huang for their interest in our paper [1, 2] and their recommendations to further refine our research.

First, we would like to point out that the discussion section of our paper explicitly addresses radiation-related risks, referring to lifetime attributable risk (LAR) estimations based on the BEIR VII Committee’s risk models, which apply a dose-response assumption consistent with the linear no-threshold (LNT) model—a widely used risk assumption based on biological considerations [3]. The LNT hypothesis is specifically applied in radiation protection, in line with the precautionary principle that advocates for minimising potential risks even at low doses where scientific evidence of harm may be limited [4, 5].

We acknowledged that, despite higher breast doses associated with digital breast tomosynthesis plus synthesised mammography (DBT + SM) compared to digital mammography (DM), the potential survival benefit due to improved cancer detection rates may lead to a favourable benefit-risk ratio for women with dense breasts [6]. As the primary objective of our study was to compare breast dose values for DM and DBT + SM across different categories of breast densities and age groups, a detailed quantitative risk analysis was beyond the trial´s intended scope.

Second, cost-effectiveness analyses are definitely required to address the potential public health implications of our findings. When conceptualising TOSYMA in 2015, the focus was still on whether it was permissible to abandon the additive concept   DBT + DM and instead use DBT + SM for screening: technological innovations are subject to rapid changes, so that, in our view, economic evaluations appear more appropriate closer to implementation in health systems. Of note, however, the introduction of artificial intelligence in image analysis is about to fundamentally change the time and resources required for the acquisition and reading of screening images already in the near future. This will also change the financial framework, making economic analyses, among others, like risk-adapted screening strategies, an indispensable part of a refocused discussion about the allocation of health resources.

Third, as already addressed above, the evaluation of hard endpoints is beyond the scope of current (and presumably also future) trials, so research is bound to put the focus on commonly accepted surrogate endpoints. TOSYMA-2 is presently evaluating interval cancer rates and has applied for an extension of data collection to assess the cumulative 2-year incidence of breast cancers in the subsequent screening round, including specifically also that of advanced cancers in the study arms.

Finally, TOSYMA is a large, pragmatic RCT embedded in an ongoing population screening programme, implying the use of mammography devices from different vendors (conditional on their preceding certification). It is correct that the radiation exposure varies between each device, model and vendor. An individual evaluation of each device was not the subject of TOSYMA. In fact, we emphasise that this approach corroborates the generalizability of our study as it shows what the mixture of different devices can achieve in a population screening—a so-called real-world approach. Based on this general approach, for example, national dose reference levels (DLR) or the LAR can be estimated for DBT. In Germany 2023, new DLR for DBT were already introduced due to the TOSMYA data.

Regarding the comparison of SM and DM, there seems to be a misunderstanding by the authors: we did not apply double exposure, but compared the established digital mammography with the DBT; the synthetic 2D image was reconstructed from the data set of the DBT without radiation exposure.

We readily join the authors in the call for future research perspectives that prioritise innovative technologies and risk-adapted screening strategies. The available data on the use of DBT + SM, including our own research, has already led to preliminary evaluations by the authorities in Germany with regard to a possible introduction into the nationwide breast cancer screening programme.
